# Mechanical strain to maxillary incisors during direct laryngoscopy

**DOI:** 10.1186/s12871-017-0442-z

**Published:** 2017-11-07

**Authors:** Milo Engoren, Lauryn R. Rochlen, Matthew V. Diehl, Sarah S. Sherman, Elizabeth Jewell, Mary Golinski, Paul Begeman, John M. Cavanaugh

**Affiliations:** 1 Department of Anesthesiology, University of Michigan, Ann Arbor, MI USA; 2 Department of Anesthesiology, Mercy St. Vincent Medical Center, Toledo, OH USA; 3 Department of Biomedical Engineering, Wayne State University, Detroit, MI USA; 4 Beaumont Graduate Program of Nurse Anesthesia, Oakland University, Rochester, MI USA

**Keywords:** Laryngoscopy, Dental injury, Strain, Intubation

## Abstract

**Background:**

While most Direct laryngoscopy leads to dental injury in 25–39% of cases. Dental injury occurs when the forces and impacts applied to the teeth exceed the ability of the structures to dissipate energy and stress. The purpose of this study was to measure strain, (which is the change produced in the length of the tooth by a force applied to the tooth) strain rate, and strain-time integral to the maxillary incisors and determine if they varied by experience, type of blade, or use of an alcohol protective pad (APP).

**Methods:**

A mannequin head designed to teach and test intubation was instrumented with eight single axis strain gauges placed on the four maxillary incisors: four on the facial or front surface of the incisors and four on the lingual or back, near the insertion of the incisor in the gums to measure bending strain as well as compression. Anesthesiology faculty, residents, and certified registered nurse anesthetists intubated with Macintosh and Miller blades with and without APP. Using strain-time curves, the maximum strain, strain rate, and strain time integral were calculated.

**Results:**

Across the 92 subjects, strain varied 8–12 fold between the 25th and 75th percentiles for all four techniques, but little by experience, while strain rate and strain integral varied 6–13 fold and 15–26 fold, respectively, for the same percentiles. Intubators who had high strain values with one blade tended to have high strains with the other blade with and without the APP (all pairwise correlation rho = 0.42–0.63).

**Conclusions:**

Strain varies widely by intubator and that the use of the APP reduces strain rate which may decrease the risk of or the severity of dental injury.

## Background

The ability to quickly, smoothly, and safely intubate the trachea is a key skill for an anesthesia provider to possess. Ideally, when using a rigid laryngoscope, the intubator gets a clear view of the larynx and inserts the endotracheal tube while avoiding contact with the maxillary teeth. However, previous studies have shown that contact is common and that the incidence of dental injury is high: 25–39% [1–4]. Injuries include avulsion and dislocation, in which the tooth is removed or loosened at the root, fractures through the dentin and pulp, and, most commonly, fractures solely to the enamel. Fractures range from visible loss of a section of the tooth to microscopic. While many of these injuries do not get repaired and may be unrecognized by the intubator, the average cost of dental repair is $2000 [5]. Dental injury occurs when the forces and impacts applied to the teeth result in strains that exceed the ability of the structures to dissipate energy and stress. The rate at which the force is applied (dF/dt) directly affects strain rate of the loaded material. Higher strain rates to biological materials result in a stiffer or more brittle response, with tissue failure occurring at lower strains compared to loading at lower rates. Forces can be applied axially as a compressive load across the whole tooth or axially localized to a portion of the tooth. Forces localized to a portion of the tooth result in compression under the force with tension to the structures immediately surrounding the compression. Forces can also be applied transversely to the tooth, producing a torque that results in stresses due to bending, with possible dislocation or fracture, particularly if there is a fulcrum.

**Fig. 1:**
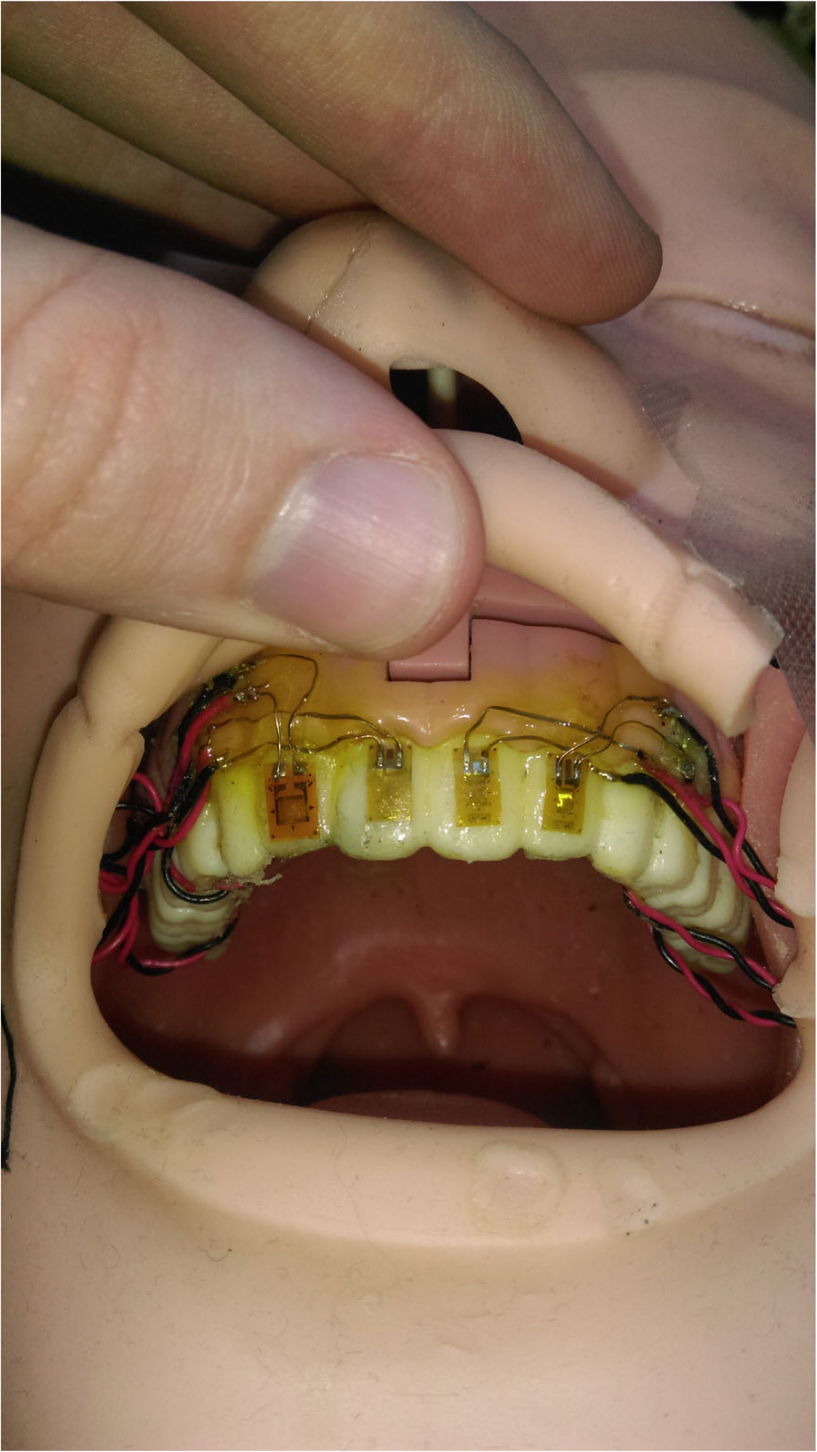
Photograph of mannequin showing strain gauges attached to the upper incisors

Studies have investigated the forces applied to the tongue and periglottic structures, [6–10] and separately, have measured forces applied to the maxillary teeth [2, 11–14]. While some studies found lower forces on the teeth with videolaryngoscopes than with Macintosh blades and some found lower forces by experienced than inexperienced intubators, most only studied axial forces [2, 11–15]. However, the major limitation of all these studies is that they only described force. While strain is the change in length divided by the original length (ΔL/L) produced by a force, (Fig. 1), strain rate may better correlate with dental injury similar to other injuries where the rate at which a force is applied or strain occurs better determines injury than the value of the force or strain itself does [16, 17]. The slower the strain rate, the more time the tissue fibers have to unfold, twist, slide, or otherwise absorb the mechanical energy without fracture [18]. Strain rate has become important in explaining both traumatic brain injury and acute lung injury [16–18]. The use of a dental protector may aid in limiting the forces applied to the teeth, and hence produce lower strain and strain rate [19].

We hypothesized that not only would inexperienced intubators place greater strain on the maxillary incisors, they would also create greater strain rate and strain-time integral. We further hypothesized that there would be little difference in strain, strain rate and strain-time integrals between Macintosh and Miller blades and that the use of a thin dental protector would cause proportionally greater decreases in strain rate than in strain or strain-time integral.

## Methods

This study was approved by the Institutional Review Boards of the University of Michigan and Wayne State University. All subjects gave consent. All faculty anesthesiologists, certified registered nurse anesthetists, and anesthesia fellows and residents were eligible. They were solicited via group emails, flyers in the lounge, and personal invitation. Participants had the study explained to them by one of the investigators and completed an anonymous demographic sheet. An instrumented intubating mannequin head (Laerdal 006257, Wappingers Falls, NY), without adjustable features, was placed on a standard operating room table, which the intubators were permitted to raise or lower to achieve their preferred heights and to position the head and neck as desired, in a climate controlled room at constant temperature. The mannequin’s teeth are polyvinyl chloride and their mechanical characteristics would not be affected by the ~500 intubations during this study (personal correspondence Laerdal Technical Support Aug 15, 2017). In random order, participants were handed a laryngoscope handle with either a Miller 3 or a Macintosh 4 blade, then the other blade, for intubation. Next, they repeated the laryngoscopy and intubation using a protective pad, an alcohol pad in its wrap, (APP) (Webcol KDL5110, Covidien, Mundelein, IL) placed on the maxillary incisors. The Miller 3 and Macintosh 4 blades were used in random order with the APP.

**Fig. 2:**
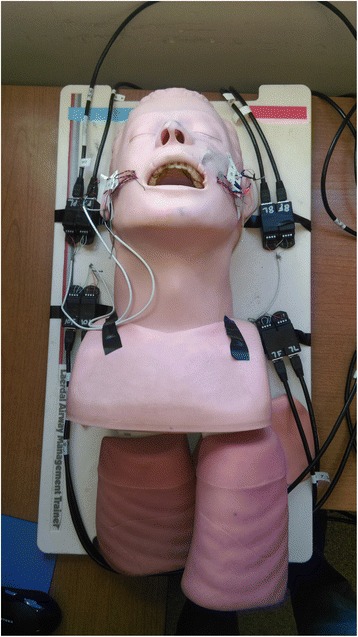
Photograph of instrumented mannequin

**Fig. 3:**
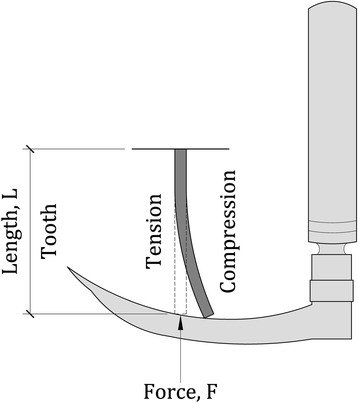
Schematic drawing showing strain, change in length divided by length (ΔL/L), which is dimensionless. If the forces producing tension and compression are the same magnitude, the magnitude of ΔL will be the same, but of opposite sign

**Fig. 4:**
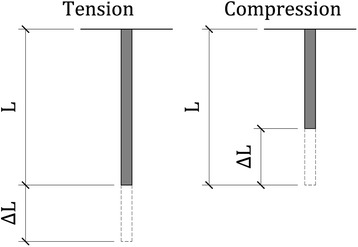
Schematic drawing showing the force, F, as the laryngoscope blade contacts the tooth of length. The blade produces tension (stretch) on one side of the tooth and compression on the other side. The amount of tension and compression are measured by strain gauges glued to each side of the tooth

The mannequin head (Figs. 1 and 2) was instrumented with eight single axis strain gauges (General Purpose Strain Gauges Linear Pattern 062AK, Micro Measurements, Raleigh, NC) placed on the four maxillary incisors: four on the front surface of the incisors and four on the back surface. These gauges were placed near the insertion of the incisor in the gums to measure bending strain as well as strain due to compression. The strain on the incisors would increase if the incisors are contacted forcefully by the laryngoscope blade. (Figs. 3 and 4) If the procedure causes inward force on the incisors, the strain gauges on the front of the teeth will go into tension and the lingual strain gauges will go into compression. If the procedure causes outward force on the incisors, the reverse will occur. If the force is primarily directed axially from the tip of the incisors toward the gums, the strain gauges will all measure compressive strain. The strain transducers were connected to an 8-channel data acquisition system (Ethernet Based Strain Measurement Module 6224, Measurement Computing, Norton, MA) with a 1000 hertz sample rate. This system was in turn was connected to a laptop computer that recorded the strain applied to the maxillary teeth and the time over which the force was applied. Strains were measured from the time of laryngoscope insertion until the endotracheal tube was placed in the larynx. The strain was measured separately for each of the strain gauges for each intubation.

While inspecting the data collected from the first day of acquisition, it was noted that strain values varied widely in both magnitude and location. Due to this observation it was hypothesized that the laryngoscope blade was not the only cause of dental strain. Other causes were the manual manipulation of the jaw by the practitioner, pressure exerted to the maxillary region, the insertion of the endotracheal tube, and impact of the dummy’s head to the operating table. To further investigate whether these events could cause discernable strain, the testing conditions were recreated in a controlled setting and the events were simulated. During this testing some of the potential strain causes were excluded but two were determined to cause significant amounts of strain: manual manipulation of the jaw and the insertion of the endotracheal tube. The data were inspected for discernable trends that would indicate the cause of each occurrence of such strain. This trend analysis found that strain caused by the laryngoscope blade was concentrated on tooth 8 (the right upper medial incisor) and that it caused primarily compressive strain in both the facial and lingual strain gauges.

Following complete data acquisition, the strain vs time data were imported into DIAdem (National Instruments, Austin, TX) for further inspection. The data from all intubations on all days of data collection were run through a prepared script to isolate only the desired information channels which contained the strain on the facial and lingual sides of the top front four incisors (strain from the blade). In addition to extracting these channels the script also processed the data to prepare it for mathematical manipulation. A low pass Butterworth filter was applied to the channels to ensure that noise would not limit the accuracy of the results. The script also ensured that the strain data were zeroed by subtracting the mean of the first 100 values from the curve to ensure that the strain vs time curves began at the origin.

In order to extract the necessary data points to determine whether either blade type or the use of an APP as a shock absorber resulted in significantly different amounts of strain, all eight strain gauge data channels were examined for all three hundred and sixty-eight trials. In total, nearly three thousand channels of data were processed. In order to determine the maximum strain in each trial, the strain vs time plots were generated for all eight data channels. From each of these plots the maximum strain magnitude was recorded. This was completed using a peak finding scripted function. A sample group of one hundred channels was also checked by hand to ensure that the script was functioning properly. A time range of ± two seconds from the maximum strain magnitude was also noted for use during the calculation for strain rate. Determining the maximum strain rate was completed manually because the criteria established for this value made it difficult to generate a coded function. This study wanted only to examine the maximum strain rate caused by the laryngoscope blade while the laryngoscopist obtains a view of the glottis for the insertion of an endotracheal tube. It was also important that these values be taken during the time of strain loading and not during the unloading of the tooth. This is to say that if the tooth underwent compression the strain rate should have a negative sign to match the loading condition. The strain vs time plots were differentiated to provide strain rate vs time plots. The above mentioned time frame of ± two seconds from peak strain was then examined and the maximum magnitude of strain rate from that time frame was taken. The maximum strain rate was only determined for the data channel that recorded the maximum strain magnitude in each trial. The strain integral was similarly only determined for the channel that underwent maximum strain for each trial. This value was found by integrating the strain vs time plots and recording the final value on the resulting plots. This final value is the area under the curve for the entire duration of the trial.

## Power analysis

As there is a lack of information on dental strain during laryngoscopy, we based our power analysis on forces generated at the epiglottis, [7] which found that experienced laryngoscopists generated 32 ± 11 N. Using an alpha = 0.05 and beta = 0.90, it would take 80 subjects to detect a 12.5% increase in intubating force to 36 ± 11 N. Because of possible differences in the forces applied to the larynx and the incisors, we planned to study 100 subjects. As strain is linearly related to force, a 12.5% increase in strain also required 100 subjects with alpha = 0.05 and beta = 0.90.

## Statistics

Demographics of subjects were described using frequencies and proportions or medians and interquartile ranges. Because strain, strain rate, and strain integral were very right skewed, the values were log transformed, described with medians and interquartile ranges, and compared with Mann-Whitney U tests. No adjustment was made for multiple comparisons. Next linear regressions of the log transformed values were done to determine the association of blade type, APP, and intubator characteristics with the strain, strain rate, and strain integral. To assess consistency of individuals across the four intubations, that is, whether some intubators were consistently good or consistently bad, the pairwise Spearman rho statistic was calculated for strain. *P* < .05 and 95% confidence intervals that excluded 0 denoted statistical significance. Statistics were done in R version 3.2.2 (R Foundation, Vienna, Austria).

## Results

Ninety-four anesthesia providers completed the demographic form, two of whom did not do any intubations and were removed from further analysis. The study was terminated short of the desired 100 participants after all interested providers participated. The participating 92 subjects were mostly male and righthanded. They were approximately equally split amongst certified registered nurse anesthetists, anesthesiology residents, and anesthesiology faculty (Table 1).

**Table 1:** Subject Characteristics

Factor	Number	Percent
Right handedness	82	89%
Male	54	59%
Position		
Resident	40	43%
CRNA^a^	29	32%
Faculty Anesthesiologist	23	25%
Preferred Blade		
Miller	8	9%
Macintosh	66	72%
No preference	18	20%
	Median	Interquartile range
Height (cm)	173	163–180
Years of experience^b^	8	5–13
Number of intubations in the previous 30 days	10	2–30

There were 13 clinical year (CA) zeroes (interns who haven’t started their intraoperative anesthesia training, 9 CA-1, 7 CA-2, 5 CA-3, 5 CA-4, and 1 CA-5

^a^includes one 2nd year nurse anesthesia student

^b^Experience (years) was calculated from the start of clinical anesthesiology training for resident and faculty anesthesiologists and the start of clinic nurse anesthesia school for certified registered nurse anesthetists

**Fig. 5:**
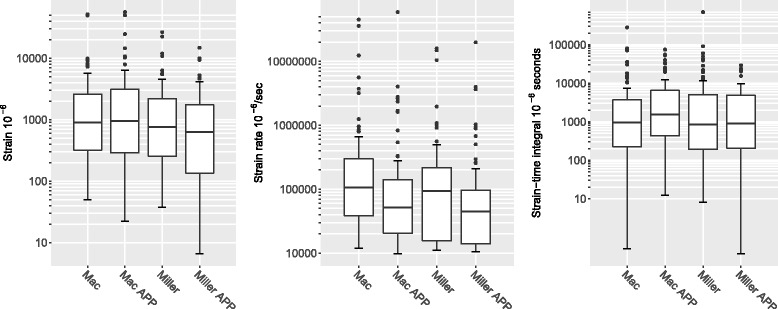
(left) Logarithmic box and whisker plot of strain (median = heavy black line, box = 1st – 3rd interquartile range, Tukey’s whiskers = 150% of the interquartile range above and below the 75th and the 25th percentiles, respectively, and dots = outliers) for Macintosh (Mac) and Miller blades. Alcohol protective pad (APP). Pairwise p’s (by MannWhitney U test): Mac v. Mac with APP (*p* = .877), Mac v. Miller (*p* = .280), Mac v. Miller with APP (*p* = .077); Mac with APP v Miller (*p* = .251), Mac with APP v, Miller with APP (*p* = .054), and Miller v. Miller with APP (*p* = .429). (center) Logarithmic box and whisker plot of strain rate (median = heavy black line, box = 1st – 3rd interquartile range, Tukey’s whiskers = 150% of the interquartile range above and below the 75th and the 25th percentiles, respectively, and dots = outliers) for Macintosh (Mac) and Miller blades. Alcohol protective pad (APP). Pairwise p’s (by MannWhitney U test): Mac v. Mac with APP (*p* = .001), Mac v. Miller (*p* = .086), Mac v. Miller with APP (*p* < .001); Mac with APP v Miller (*p* = .144), Mac with APP v, Miller with APP (*p* = .245), and Miller v. Miller with APP (*p* = .009). (right) Logarithmic box and whisker plot of strain time integral (median = heavy black line, box = 1st – 3rd interquartile range, Tukey’s whiskers = 150% of the interquartile range above and below the 75th and the 25th percentiles, respectively, and dots = outliers) for Macintosh (Mac) and Miller blades. Alcohol protective pad (APP). Pairwise p’s (by MannWhitney U test): Mac v. Mac with APP (*p* = .110), Mac v. Miller (*p* = .898), Mac v. Miller with APP (*p* = .988); Mac with APP v Miller (*p* = .179), Mac with APP v, Miller with APP (*p* = .100), and Miller v. Miller with APP (*p* = .880)

Strain was similar among groups, ranging from [median (interquartile range)] 6.3 × 10^−4^ (1.3–17.4 × 10^−4^) for Miller blade with APP to 9.5 × 10^−4^ (2.9–31.3 × 10^−4^) for Macintosh blade with APP (Fig. 5 left). However, there was a very large spread of strains produced by both the Miller and the Macintosh blades, which did not differ by type of blade or use or nonuse of the APP. Even within the middle two quartiles (25th to 75th percentile), strain varied widely, approximately 10-fold for all four techniques. Intubators who had high strain values with one blade with or without the APP tended to have high strains with the other blade with and without the APP (all pairwise rho = 0.42–0.63).(Table 2) However, after adjustment for other factors, the use of the APP with the Miller blade was associated with a slightly lower strain, Log(strain) (B = −0.218, 95% confidence interval = −0.413 to −0.024, *p* = .029 compared to the Macintosh blade). (Table 3, top).

**Table 2:** Correlation coefficients of strain by the laryngoscopists

	Macintosh	Miller	Macintosh - APP	Miller - APP
Macintosh	1	0.50	0.47	0.42
Miller	0.50	1	0.51	0.56
Macintosh - APP	0.47	0.51	1	0.63
Miller - APP	0.42	0.56	0.63	1

Correlation coefficients (Spearman’s rho) between the strains generated by the same larygnoscopist using the different blades and techniques. *APP* alcohol protective pad. All p < .001

**Table 3:** Factors associated with strain, strain rate, and strain-time integral using multivariable linear regression

	Estimate	95% Confidence Interval	*p*-value
Log Strain			
Male	−0.258	−0.415, −0.101	0.001
Position			
Faculty	0		
CRNA^a^	−0.281	−0.490, −0.072	0.009
Resident	0.163	−0.043, 0.368	0.122
Experience (years)^b^	0.010	−0.002, 0.021	0.103
Blade			
Mactinosh	0		
Miller	−0.107	−0.301, 0.087	0.282
Macintosh APP	0.013	−0.181, 0.207	0.896
Miller APP	−0.218	−0.413, −0.024	0.028
Log strain rate			
Height (cm)	−0.023	−0.042, −0.003	0.024
Blade			
Macintosh	0		
Miller	−0.179	−0.385, 0.027	0.089
Macintosh APP	0.283	−0.489, −0.077	0.007
Miller APP	−0.399	−0.605, −0.194	<0.001
Log Strain-time Integral			
Male	−0.423	−0.649, −0.197	<.001
Position			
Faculty	0		
CRNA^a^	−0.268	−0.570, 0.033	0.082
Resident	0.161	−0.162, 0.449	0.273
Experience (years)	0.015	−0.001, 0.031	0.073
Blade Preference			
Macintosh	0		
Miller	0.465	0.094, 0.836	0.015
No preference	−0.046	−0.287, 0.196	0.712

Table shows the adjusted effect of each item on the amount of log strain, log strain rate, and log strain-time integral using Akaike Information Criteria and multivariable linear regression. For categorical variables, the estimate is the amount that variable increase (or decreases) the log strain, log strain rate, and log strain-time integral by. For continuous variables, the estimate is the amount that one unit of that variable will increase (or decrease) log strain, log strain rate, and log strain-time integral by

^a^includes one 2nd year nurse anesthesia student

^b^Experience (years) was calculated from the start of clinical anesthesiology training for resident and faculty anesthesiologists and the start of clinic nurse anesthesia school for certified registered nurse anesthetists

Strain rates were similar between the Miller and Macintosh blades, both with and without the APP. However, using the APP was associated with a halving of the strain rate for each blade, *p* = .009 for the Miller blade and, *p* = .001 for the Macintosh blade. (Fig. 5 center) After adjustment for other factors, both the Miller blade with APP and Macintosh blade with APP were associated with lower Log(strain/time). (Table 3, middle).

Strain-time integral did not differ between blades with or without the APP. (Fig. 5 right) After adjustment for other factors, only users’ preference for a Miller blade was associated with an increased strain integral, B = 0.465 × 10^−6^ s (95% confidence interval = .094–0.836 × 10^−6^), *p* = .015. (Table 3, bottom).

## Discussion

We found that there was a very wide variation in strains, with many-fold variation between participants. There was little to no differences in strain by training, years of experience, or number of intubations in the previous month. We also found that the use of the APP was associated with a no change in strain or strain integral, but a 31% reduction in strain rate.

Teeth contain an inner layer of pulp, which has nerves and blood vessels, surrounded by a layer of dentin, which is then surrounded by a hard outer coating of enamel, and is anchored to the alveolar bone by the periodontal ligament, which helps to stabilize the tooth. Enamel is a mineral and organic composite. The mineral phase primarily consists of calcium phosphate salts (P_2_O_5_ and CaO) in the form of hexagonal hydroxyapatite crystals that are oriented to form rod-like structures called prisms. These prisms are separated from each other by a thin organic sheath, consisting mostly of water and protein. The molecular makeup of hydroxyapatite varies by location within the enamel, having less P_2_O_5_ and CaO and more Na_2_O and MgO as the enamel approaches the softer enamel-dentine junction [20]. There are also microscopic variations in hardness and stiffness within the tooth, with lingual surfaces being harder than labial surfaces and chewing surfaces being harder than the tooth face [20]. Other processes, such as caries, fillings, and even bleaching or whitening agents weaken the enamel [21, 22]. Enamel is harder and more brittle, hence more likely to fracture than dentin. Dentin has higher stress, strain, and elastic modulus to mechanical compression, making it better able than enamel to absorb forces [23]. However, as the dentin is surrounded by enamel and any load, such as a laryngoscope blade, applied to the teeth, is first applied to the enamel, adequate time must be present to allow the dentin to dissipate the force or the enamel may fracture. Hence, the same force applied faster or slower, which is measured as strain rate, is more or less likely to damage enamel, depending on whether or not there is sufficient time for the dentin to absorb the force.

We measured strain. Strain, ε, is the fraction of length (ΔL/L) (Fig. 3) that an object changes in response to a force or a pressure (force/area), ε = change in length/original length = force/(area * modulus of elasticity). The mechanical effect or damage produced by a force depends not just on the characteristics of the receiving structure but the area over which the force is spread, i.e., a given force can cause more damage by being spread over a smaller area (higher pressure). When applying a laryngoscope to the incisors, the blade may be angled on the tooth or the tooth may have a non-smooth crown, leading to vastly different pressures, for the same force, on different areas of the tooth. By measuring strain, we measure the effect of the force and pressure on the tooth. We found that using the APP with a Miller blade showed less strain than a Macintosh blade (Table 3 top, Fig. 5 left) and it reduced strain rate for both the Miller and Macintosh blades (Table 3 middle, Fig. 5 center). Rapid changes in strain may not allow sufficient time for forces to be adequately dispersed before they cause damage. APP acts like a car’s air bag. In an accident, the bag deploys, slowing the deceleration of the driver or passenger. The person still undergoes the same change in velocity, but it is spread over a longer time and, hence, decreases the chances of or the severity of injury. The APP acted to spread the strain over a longer time, thus reducing strain rate. APP can also spread the force over a larger area, thus reducing the pressure. The alcohol pad serving as a protective device has the advantages of being inexpensive and convenient – readily found in almost every operating room. However, research should be done to evaluate if other protective devices can better protect teeth.

The integral of strain with respect to time is proportional to impulse (force x time). Impulse is transferred from the intubator to the teeth. If the teeth are not anchored firmly, a significant impulse can avulse them. More commonly, the teeth are well anchored and the strain-time integral represents using the teeth as a fulcrum to further open the mouth or extend the head. During laryngoscopy, the intubator places the distal tip of the blade in the pharnyx and lifts the handle. Because the lifting force is along the axis of the handle, while the counteracting force is where the blade presses on the pharynx and is parallel to but several centimeters forward of the handle, this produces a torque (bending moment) – usually generated by the wrist, which the intubator needs to counteract in order to view the glottis. Placing the blade on the maxillary incisors and using them as a fulcrum may not only improve the view but may also decrease the torque required to view the glottis, [2] however, placing the blade on the teeth has the potential to cause dental damage.

We also found that intubators who had high strain with one blade had high strain with the other blade, with or without APP (Table 2). A previous study has shown that intubating forces are relatively consistent for the same intubator using the same Macintosh 3 blade in the same patient [8]. Our study extends this, by showing that intubators are consistently good or bad with different blades. An instrumented mannequin, similar to the one utilized in the current study, may also serve to provide immediate feedback to intubators as part of their learning this important skill. Studies will be needed to determine if instrumented mannequin simulators can effectively improve intubators’ skills and decrease dental injury.

Strain and strain rate varied more than 10-fold amongst intubators. Similar wide variations in force have been reported in intubation studies measuring force [2, 24, 25]. Study is needed to determine why strain and strain rate vary so much and what can be done to bring all intubators to the best practice of least strain.

We found that the APP, on average, reduced strain rate with both blades; there was a wide variation in intubators’ changes in strain rate between using or not using the APP, with some intubators achieving greater strain rate with APP than without. While overall, its use may be beneficial, further study or perhaps use of simulation training may be needed to help all intubators decrease the strain rate using an APP.

There are several limitations to our study. While we measured strain resulting both from forces along the axis (from crown to root) and transverse bending of the incisors (forwards-backwards), we cannot tell in any individual how much of the strain was applied in each direction. However, in actual human incisors the resultant strain is the strain of the enamel crystals making up the tooth surface, although there may be differences in the amount of strain due to axial forces and due to bending, depending on the applied force and fulcrum. Another limitation is that we used a mannequin and not patients. However, gluing strain gauges to patients’ teeth is not a practicable method in our practice and instrumenting laryngoscope handles and blades may not localize the forces or their directions. The mannequin also only provided one condition for intubating. Being able to adjust the mannequin to produce a variety of conditions such as an anterior airway, buck or missing teeth, and reduced mouth opening may produce different results. Children or edentulous patients may also have different results. Unlike several other studies, but similar to one, we found little to no effect by type of training, experience, or number of intubations in the recent past [10–12]. Perhaps our mannequin represented an easy intubation and that simulation scenarios of more difficult intubations would show differences by training or experience or show different effects by type of blade or APP use. Further study is needed to determine this.

## Conclusions

In conclusion, we found that strain varies widely by intubator and that intubators with high strain with one blade tended to have high strain with the other blade, suggesting that there may be opportunities for implementation of best practices to reduce laryngoscopy forces on the teeth. The use of an APP reduces strain rate with both Miller and Macintosh blades, but reduces strain only with Miller blades. Its use may decrease dental injury.

## Abbreviation

APP: alcohol protective pad
